# Dietary l-arginine Supplementation Alleviates the Intestinal Injury and Modulates the Gut Microbiota in Broiler Chickens Challenged by *Clostridium perfringens*

**DOI:** 10.3389/fmicb.2018.01716

**Published:** 2018-07-31

**Authors:** Beibei Zhang, Zengpeng Lv, Zhui Li, Weiwei Wang, Guang Li, Yuming Guo

**Affiliations:** State Key Laboratory of Animal Nutrition, College of Animal Science and Technology, China Agricultural University, Beijing, China

**Keywords:** l-arginine, gut microbiota, *Clostridium perfringens*, PICRUSt predicted functions, 16S rRNA high-throughput sequencing, broiler chicken

## Abstract

Our previous reports suggested that Dietary l-arginine supplementation attenuated gut injury of broiler chickens infected with *Clostridium perfringens* by enhancing intestinal immune responses, absorption and barrier function, but its effect on the gut microbiome of broiler chickens remains unclear. This experiment aimed at evaluating the effects of Dietary l-arginine supplementation on the gut bacterial community composition and function of broiler chickens challenged with *C. perfringens*. In total, 105 1-day-old male Arbor Acres broiler chickens were assigned to three groups: Control (CTL), *C. perfringens-*challenged (CP), and *C. perfringens*-challenged and fed diet supplemented with 0.3% l-arginine (ARGCP) groups. The challenge led to macroscopic and histomorphological gut lesions, decreased villus height and increased the number of Observed species, Shannon, Chao1 and ACE indices of ileal microbiota, whereas l-arginine addition reversed these changes. Moreover, the three treatments harbored distinct microbial communities (ANOSIM, *P* < 0.05). At the genus level, 24 taxa (e.g., *Nitrosomonas* spp., *Coxiella* spp., *Ruegeria* spp., and *Thauera* spp.) were significantly more abundant in CP group than in CTL group (*P* < 0.05), whereas the levels of 23 genera of them were significantly decreased by l-arginine supplementation (*P* < 0.05). The abundances of only 3 genera were different between CTL and ARGCP groups (*P* < 0.05). At the species level, the challenge promoted the relative abundance of *Nitrospira* sp. *enrichment culture clone* M1-9, *Bradyrhizobium elkanii, Nitrospira bacterium* SG8-3, and *Pseudomonas veronii*, which was reversed by l-arginine supplementation (*P* < 0.05). Furthermore, the challenge decreased the levels of *Lactobacillus gasseri* (*P* < 0.05). Predictive functional profiling of microbial communities by PICRUSt showed that compared with CP group, ARGCP group had enriched pathways relating to membrane transport, replication and repair, translation and nucleotide metabolism and suppressed functions corresponding to amino acid and lipid metabolisms (*P* < 0.05). The relative abundances of KEGG pathways in l-arginine-fed broilers were almost equal to those of the controls. In conclusion, l-arginine alleviated the gut injury and normalized the ileal microbiota of *C. perfringens*-challenged chickens to resemble that of unchallenged controls in terms of microbial composition and functionality.

## Introduction

Necrotic enteritis (NE) is a universal enteric bacterial disease in poultry and is related to extensive production losses worldwide (Wade and Keyburn, 2015). The etiologic agent, *Clostridium perfringens* is a spore-forming, gram-positive anaerobe and is a normal inhabitant of the intestinal tract in healthy birds (Cooper and Songer, 2009). However, under certain pre-disposing conditions, this bacterium can proliferate to high numbers and meanwhile produce extracellular toxins, causing an outbreak of NE (Van Immerseel et al., 2009). NE can present as clinical and subclinical conditions. Clinical symptoms of this disease are characterized by severe necrosis of intestine and high flock morbidity and mortality, whereas the subclinical infection causes intestinal mucosal damage with no mortality, thus led to poor nutrient digestion and absorption, and further impaired growth performance (Van Immerseel et al., 2009; Timbermont et al., 2011).

Emerging evidence has shown that NE is strongly associated with intestinal microbiota in broiler chickens. There are strikingly varied microbial compositions in the onset and progression of NE (Stanley et al., 2012; Antonissen et al., 2016). For instance, *C. perfringens* challenge significantly reduces the level of *Weissella confusa* but significantly increases the abundance of uncultured Mollicutes related to human problems in chickens (Stanley et al., 2012). Reduction of *Lachnospiraceae* and *Ruminococcaceae* levels and the promotion of *Gammaproteobacteria* by NE have also been reported (Li et al., 2017). However, whether these shifts in microbiota communities are the results of other pre-disposing factors or whether they are more a consequence of *C. perfringens* multiplication and necrosis is still unclear.

A shift in diet can quickly affect the composition of the gut microbial community (David et al., 2014). Studies have suggested that dietary administration can affect the population of *C. perfringens* in the intestine (Liu et al., 2010) and influence the occurrence of this disease (Timbermont et al., 2011). With the removal of growth-promoting antibiotics, nutritional strategies are becoming a potential intervention for NE.

l-arginine is an essential amino acid for chickens and can be metabolized to produce important molecules such as nitric oxide, polyamines and creatine. l-arginine has presented great benefits in protecting intestinal health in numerous disease models. Tan et al. (2014a) reported that arginine-enriched diets alleviated intestinal villus damage, crypt dilation and goblet cell depletion induced by coccidia in broiler chickens. Moreover, these diets played a protective role in mice subjected to exertional hyperthermia by decreasing intestinal permeability and bacterial translocation (Costa et al., 2014). Furthermore, our results have recently shown that Dietary l-arginine addition protects the gut mucosa by improving innate immune responses, intestinal absorption and barrier function and by suppressing the colonization of *C. perfringens* in necrotic enteritis-challenged broiler chickens (Zhang et al., 2017).

Recent findings suggest that l-arginine regulates microbial composition and metabolism. Zheng et al. (2017) demonstrated that dentifrice supplemented with arginine favorably modulated the oral microbiome of individuals with dental caries, which was characterized by increasing the abundance of alkali-generating bacteria and reducing the content of acidogenic bacteria. With l-arginine addition, net utilization of alanine, glycine, threonine, lysine, leucine and isoleucine by the small-intestine bacteria of pig was reduced *in vitro* (Dai et al., 2012). However, to our knowledge, no studies have reported the function of l-arginine in the homeostasis of ileal microbial communities in broiler chickens. This study was undertaken to investigate the effects of arginine-supplemented diets on ileal microflora composition and function in broiler chickens challenged with *C. perfringens* by using 16S rRNA gene sequencing.

## Materials and methods

### Experimental animals, diets, and treatments

This study was approved by the China Agricultural University Animal Care and Use Committee (statement no. CAU20170601-2). A total of 105 1-day-old male Arbor Acres broiler chickens were randomly divided into three groups: Control (CTL), *C. perfringens*-challenged (CP), and *C. perfringens*-challenged and fed diet supplemented with 0.3% l-arginine (ARGCP) groups. Each group, involving 35 birds, was housed in a separate rearing isolator (160 × 70 × 70 cm) in a common room with controlled temperature. The experimental period lasted 21 days. All chickens were provided with feed and water *ad libitum* and received a 23 h light-1 h dark program every day. In addition, the chickens were vaccinated according to a routine immunization program. Corn-soybean meal diets were formulated according to the nutrient requirements for broilers as recommended by National Research Council (1994). The diet composition and nutrient levels are shown in Table S1. All diets were pelleted and crumbled. Dietary amino acid contents were determined by HPLC.

### *C. perfringens* challenge

Avian *C. perfringens* type A field strain (CVCC2030), received from the China Veterinary Culture Collection Center (Beijing, China), has been successfully used to establish NE model in broilers in our lab (Li et al., 2017). Briefly, chickens in the CP and ARGCP groups were infected with 1.0 mL of actively growing culture of *C. perfringens* at 2~3 × 10^8^ CFU/mL by oral gavage each day from day 14 to day 20. Birds in the CTL group received an equal volume of sterile meat medium.

### Sample collection

On d 21, seven chickens per group were randomly selected and intravenously injected into pentobarbital sodium in a dose of 30 mg/kg body weight. Then these chickens were sacrificed by jugular exsanguination. The mid regions of the jejunum (~1 cm) were cut off and the digesta inside were gently rinsed with ice-cold saline. Then, the jejunal rings were immediately fixed in 4% paraformaldehyde for further intestinal morphological examination. ~2 g of digesta from the midpoint of the ileum was aseptically collected into sterile tubes, snap-frozen in liquid nitrogen, and stored at −80°C for DNA extraction.

### Intestinal lesion score

The small intestine of each chicken was cut longitudinally and scored blindly as described by Dahiya et al. (2005) with some modifications. Lesions were evaluated with a scoring system from 0 to 4.0 = no gross lesions; 0.5 = severely congested serosa and mesenteric hyperemia; 1 = thin-walled and brittle intestines with small hemorrhagic spots (>5); 2 = small amounts of gas production and focal necrotic lesions 3 = gas-filled intestine and necrotic plaques (1–2 cm long); and 4 = large amounts of gas in the intestine and diffused necrosis.

### Intestinal morphological analyses and observation

The jejunum segments fixed in 4% paraformaldehyde were embedded in paraffin. Tissue rings were cut to a thickness of 5 μm and stained by hematoxylin and eosin. The slides were photographed by a Leica microscope (Wetzlar, Germany, Model DMi8). Villus height and crypt depth were measured from eight villi and crypts per slide by a blinded viewer using the Image-Pro Plus (version 6.0) software and averaged. Villus height was defined as the distance from the villus tip to the villus-crypt junction, and the crypt depth was measured from the villus-crypt junction to the base of the crypt. The means of villus height and crypt depth were calculated to obtain the villus height-to-crypt depth ratio (VCR). Morphological analyses and observation were conducted at magnifications of 50 × for each slide.

### DNA extraction and high-throughput sequencing

Bacterial DNA was extracted from ileal digesta with a QIAamp DNA Stool Mini Kit (Qiagen Inc., Valencia, CA) according to the manufacturer's protocol. The concentrations of DNA extracts were measured on a NanoDrop 2000 spectrophotometer (Thermo Scientific, MA, USA). The V4 region of the bacterial 16S rRNA gene was amplified with the barcoded primer pair 515F/806R (515F: 5′-GTG CCA GCM GCC GCG GTA A-3′, 806R: 5′-GGA CTA CHV GGG TWT CTA AT-3′) according to previously described methods (Wang et al., 2017). After amplification, PCR products run on a 2% agarose gel and were purified using a QIAquick Gel Extraction Kit (Qiagen, Germany). Pyrosequencing of 16S rDNA was performed on an Illumina HiSeq2500 PE250 platform (Illumina, San Diego, USA) at Novogene Bioinformatics Technology Co. Ltd. (Beijing, China).

### Sequence processing and bioinformatics analysis

Raw tags were generated by merging paired-end reads using FLASH software (v1.2.7) (Magoc and Salzberg, 2011). High-quality clean tags were obtained by QIIME (v1.7.0) analysis (Caporaso et al., 2010), and chimera sequences were removed to obtain effective tags by using the UCHIME algorithm (Edgar et al., 2011). Sequences were analyzed by UPARSE software (v7.0.1001) and clustered into operational taxonomic units (OTUs) at a similarity level of 97% (Edgar, 2013). Each OTU was annotated with the Greengenes database (DeSantis et al., 2006). Rarefaction curve and Venn diagram were created using R software (v2.15.3). Analysis of microbial alpha diversity was conducted using QIIME software (Caporaso et al., 2010) with Python scripts. Beta diversity was evaluated by principal component analysis (PCA) to show the differences of bacterial community structures, and the significance of separation was tested via ANOSIM using R (v2.15.3). PICRUSt analysis was used to predict the functional potential of bacteria communities (Langille et al., 2013). OTUs were normalized by copy number, and metagenome prediction was further categorized into Kyoto Encyclopedia of Genes and Genomes (KEGG) at levels 2 and 3 (Kanehisa et al., 2012).

### Statistical analysis

Results are shown as the mean and SEM. Differences in the intestinal lesion score, jejunal morphology, alpha diversity indices, the relative abundance of top-10 phyla, and the bacterial functional pathways were analyzed by one-way ANOVA, followed by the Duncan multiple comparison tests (SPSS, version 18.0, Chicago, IL, USA). Comparisons of the relative abundances of genera and species and the *Firmicutes/Bacteroidetes* ratio between 2 groups were performed by unpaired Student's *t*-test. Significant difference was declared when *P* < 0.05.

### Accession number

The 16S rRNA gene amplicon sequencing results were submitted to the Sequence Read Archive of the NCBI (accession number: SRP134059).

## Results

### Intestinal lesion score

In our study, no intestinal lesions occurred in CTL birds, except that slight congestion appeared in the intestine of 2 birds. In the CP group, all birds exhibited macroscopic gut lesions such as thin-walled intestines, focal hemorrhagic lesions and small amounts of gas production. The average lesion score was significantly lower in the challenged birds fed l-arginine-enriched diets than in the challenged birds (*P* < 0.05, Table 1). No mortality was observed in the unchallenged chickens, but *C. perfringens* challenge caused one bird's death in each of the CP and ARGCP groups.

**Table 1:** Intestinal lesion score and jejunal morphology of broilers.

**Treatment**	**Lesion score**	**VH(μm)**	**CD(μm)**	**VCR**
CTL	0.14 ± 0.092^a^	911.79 ± 35.44^b^	121.42 ± 8.05	7.65 ± 0.448
CP	1.57 ± 0.202^b^	723.19 ± 56.30^a^	101.34 ± 7.61	7.21 ± 0.439
ARGCP	0.36 ± 0.180^a^	882.36 ± 62.21^b^	109.92 ± 7.76	7.93 ± 0.608
*P*-value	< 0.001	0.038	0.217	0.607

a, b *Values within a column followed by different superscript letters differ significantly (P < 0.05). CTL, non-challenge control; CP, C. perfringens-challenged group; ARGCP, C. perfringens-challenged group fed diet supplemented with 0.3% l-arginine. VH, villus height; CD, crypt depth; VCR, the ratio of VH:CD. Data are shown as the mean ± standard error*.

### Intestinal morphology

**Figure 1:**
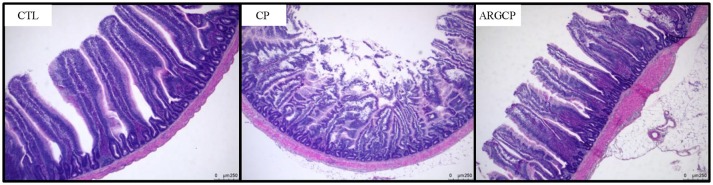
Representative photomicrographs of the jejunal cross section in chicken. Original magnification is 50 ×. CTL demonstrates the physiological features of the intestinal villus, whereas CP shows severe pathological changes affecting the normal villus architecture. ARGCP illustrates mild pathological changes with defects in a small number of epithelial cells. CTL, non-challenge control; CP, *C. perfringens*-challenged group; ARGCP, *C. perfringens*-challenged group fed diet supplemented with 0.3% l-arginine.

As shown in Table 1, the villus height in the jejunum in the CP group was significantly lower than that in the CTL and ARGCP groups (*P* < 0.05). There was no significant difference between the ARGCP group and the CTL group villus heights (*P* > 0.05). Neither *C. perfringens* challenge nor l-arginine addition affected the crypt depth and villus height/crypt depth ratio in the jejunum (*P* > 0.05). As depicted in Figure 1, control birds showed the normal appearance of the intestinal villus, whereas *C. perfringens* challenge led to severe pathological changes with the disappearance of the normal villus architecture. The ARGCP group exhibited mild pathological changes with defects of a small number of epithelial cells.

### The quality of sequencing data

After OTUs were assigned and chimeras were removed, sequencing of 21 samples generated 1,538,607 effective sequences with an average of 73,267 ± 2,356 (mean ± standard error) sequences per sample (Table S2). The median read length was 253 base pairs (bp), with a range from 253 to 255 bp (Table S2). A total of 3,597 OTUs were identified based on >97% sequencing similarity. Wherein 536 OTUs were common in three groups, and 96, 1,438, and 123 OTUs were exclusive in CTL group, CP group and ARGCP group, respectively (Figure S1). The Good's coverage estimators (Table S2) and the rarefaction curves (Figure S2) indicated that sufficient sequencing coverage was achieved.

### The alpha and beta diversity of gut microbiota

**Figure 2:**
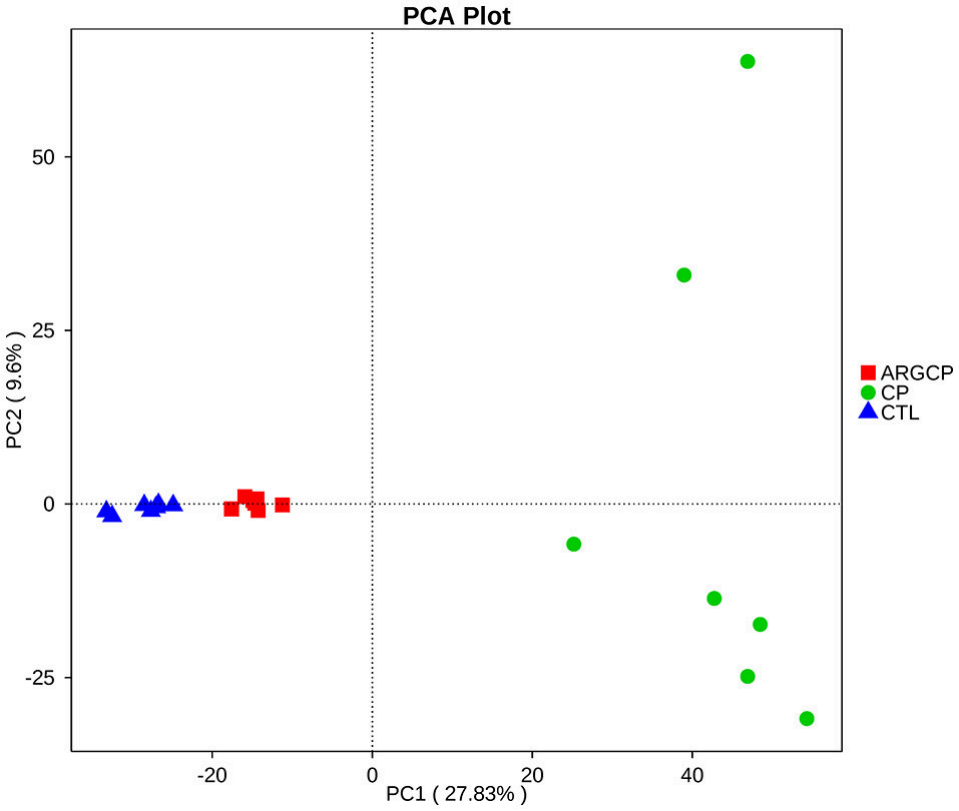
Comparison of the compositions of the ileal microbiota by principal component analysis (PCA). CTL, non-challenge control; CP, *C. perfringen*s-challenged group; ARGCP, *C. perfringens*-challenged group fed diet supplemented with 0.3% l-arginine.

The alpha diversity of ileal microbiota was shown in Table 2. Compared with CTL group, both richness indices (observed species, Chao1 and ACE) and diversity indices (Shannon) significantly increased in the CP group (*P* < 0.001). However, these indices significantly decreased in ARGCP group compared with CP group (*P* < 0.001). Beta diversity analysis was illustrated via PCA in Figure 2, showing notable differentiation of the microbial community structure among the three groups. The composition of the microbiota in the ARGCP group was more similar to that in the CTL group than that in the CP group. ANOSIM results (*P* < 0.05) indicated that these significant differences in the microbial community structure depended on the three different treatments (Table 3).

**Table 2:** The alpha diversity of ileal microbiota.

**Treatment**	**Observed species**	**Shannon**	**Simpson**	**Chao1**	**ACE**
CTL	413.14 ± 34.99^a^	3.36 ± 0.26^a^	0.81 ± 0.03	497.33 ± 38.64^a^	513.47 ± 39.84^a^
CP	1748.43 ± 44.53^c^	4.97 ± 0.23^b^	0.80 ± 0.04	2168.13 ± 90.76^c^	2255.53 ± 76.13^c^
ARGCP	816.86 ± 58.06^b^	3.46 ± 0.20^a^	0.77 ± 0.03	1098.40 ± 78.02^b^	1172.03 ± 78.57^b^
*P*-value	< 0.001	< 0.001	0.677	< 0.001	< 0.001

a, b, c *Values within a column followed by different superscript letters differ significantly (P < 0.05). CTL, non-challenge control; CP, C. perfringens-challenged group; ARGCP, C. perfringens-challenged group fed diet supplemented with 0.3% l-arginine. VH, villus height; CD, crypt depth; VCR, the ratio of VH:CD. Data are shown as the mean ± standard error*.

**Table 3:** Comparison of similarities in microbiota composition between the three treatments by ANOSIM analysis.

**Treatment**	***R*-value**	***P*-value**
CTL-CP	0.6735	0.002
CP-ARGCP	0.3635	0.009
CTL-ARGCP	0.2828	0.021

*The value of R ranges from −1 to 1. The groups are significantly different when the R-value is >0, and the intra-group difference is greater than the inter-group difference when the R-value is < 0. P < 0.05 indicates a significant difference. CTL, non-challenge control; CP, C. perfringens-challenged group; ARGCP, C. perfringens-challenged group fed diet supplemented with 0.3% l-arginine*.

### Relative abundances of the dominant phyla

The most abundant (top 10) phyla of bacteria are presented in Table 4 and Figure S3. At the phylum level, the ileal microbiota was dominated by *Firmicutes* (53~91%), *Cyanobacteria* (1~34%), *Proteobacteria* (3~33%) and *Bacteroidetes* (0.1~8%), together making up over 88% of the total sequences. The *C. perfringens*-challenged birds (CP group) had markedly lower *Firmicutes* and *Cyanobacteria* abundances than the control chickens (*P* < 0.05). However, the relative abundance of *Firmicutes* was enhanced in the ARGCP group compared with that in the CP group. Moreover, *C. perfringens* challenge resulted in significantly greater abundances of *Proteobacteria, Actinobacteria, Planctomycetes, Verrucomicrobia, Chloroflexi, Nitrospirae*, and *Acidobacteria* in the CP group than in unchallenged birds (*P* ≤ 0.001); however, l-arginine addition significantly reversed these changes found in the CP group (*P* ≤ 0.001). Little difference was observed in the relative abundances of *Firmicutes, Cyanobacteria* and *Proteobacteria* in the ARGCP and CTL groups (*P* > 0.05), and no differences were shown in the relative abundances of *Bacteroidetes* among the three treatments (*P* > 0.05). Moreover, the *Firmicutes/Bacteroidetes* ratio tended to be greater in the CP group than in the CTL group (*P* = 0.083), but l-arginine addition remarkably increased this ratio (*P* < 0.05, Figure S4).

**Table 4:** The top 10 phyla in the ileal microbiota of broilers.

**Treatment**	**Fir**	**Cya**	**Pro**	**Bac**	**Act**	**Pla**	**Ver**	**Chl**	**Nit**	**Aci**
CTL	78.08 ± 4.35^b^	9.36 ± 2.31^b^	5.83 ± 0.85^a^	2.22 ± 1.06	0.17 ± 0.04^a^	0.50 ± 0.08^b^	0.01 ± 0.003^a^	0.02 ± 0.004^a^	0.01 ± 0.002^a^	0.01 ± 0.006^a^
CP	62.00 ± 3.07^a^	3.15 ± 1.23^a^	21.21 ± 2.27^b^	3.22 ± 0.46	2.11 ± 0.11^c^	1.19 ± 0.10^c^	0.91 ± 0.07^c^	0.83 ± 0.08^c^	0.95 ± 0.06^c^	0.94 ± 0.03^c^
ARGCP	78.39 ± 2.63^b^	5.12 ± 0.99^ab^	11.70 ± 3.37^a^	1.35 ± 0.62	1.17 ± 0.16^b^	0.05 ± 0.01^a^	0.18 ± 0.03^b^	0.20 ± 0.02^b^	0.16 ± 0.01^b^	0.38 ± 0.04^b^
*P*-value	0.004	0.044	0.001	0.244	< 0.001	< 0.001	< 0.001	< 0.001	< 0.001	< 0.001

a, b, c *Values within a column followed by different superscript letters differ significantly (P < 0.05). Fir, Firmicutes; Cya, Cyanobacteria; Pro, Proteobacteria; Bac, Bacteroidetes; Act, Actinobacteria; Pla, Planctomycetes; Ver, Verrucomicrobia; Chl, Chloroflexi; Nit, Nitrospirae; Aci, Acidobacteria. CTL, non-challenge control; CP, C. perfringens-challenged group; ARGCP, C. perfringens-challenged group fed diet supplemented with 0.3% l-arginine. Data are shown as the mean ± standard error*.

### Differentially abundant gut bacterial genera

**Figure 3:**
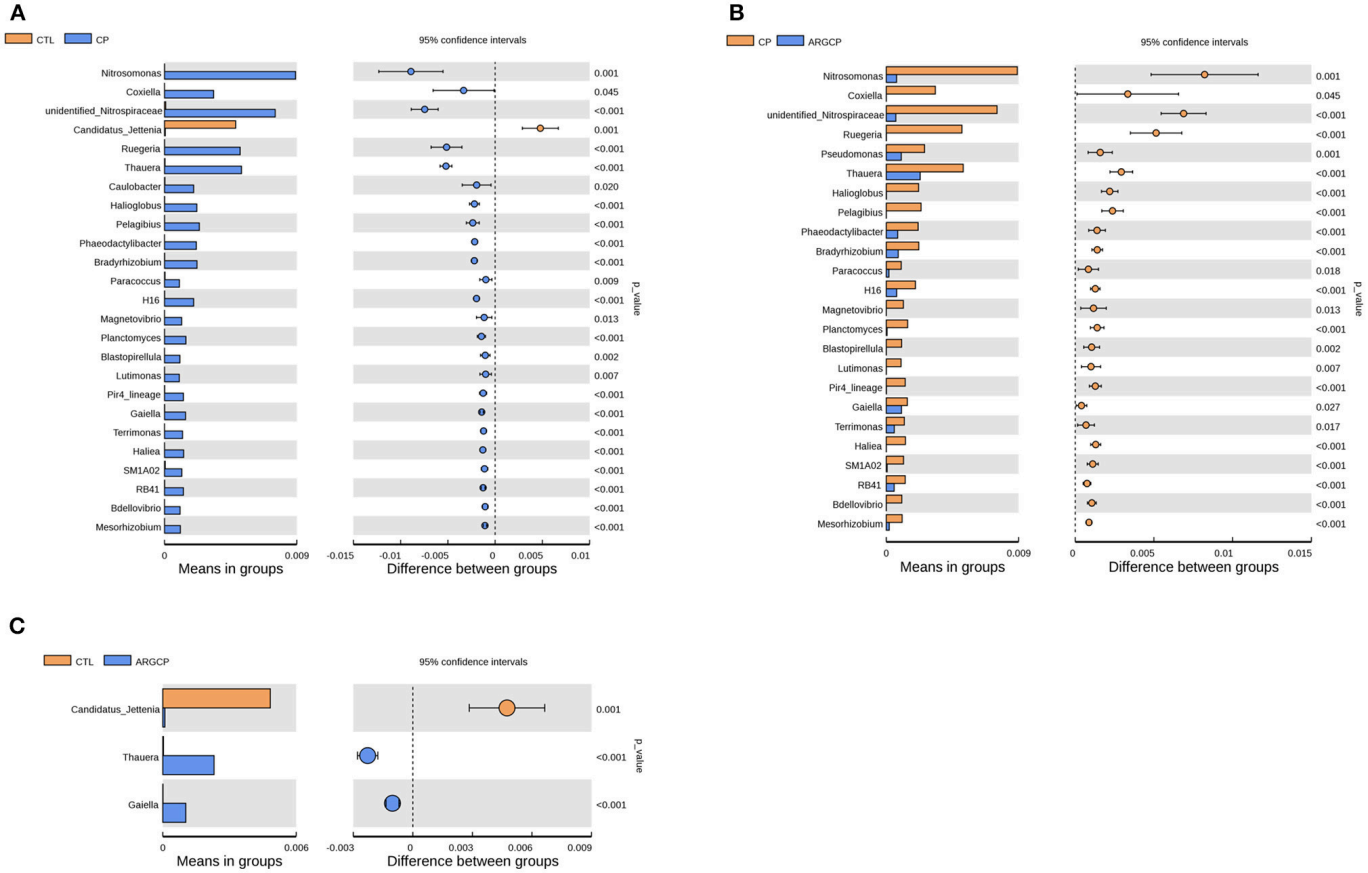
The genera differentially abundant between three treatments in the ileum by *t*-test analysis. These figures illustrate the differences in the microbiota in terms of the relative abundances of genera between groups CTL and CP **(A)**, groups CP and ARGCP **(B)**, and groups CTL and ARGCP **(C)**. Only data whose differences with *P*-values lower than 0.05 and the relative abundances higher than 0.1% in either of the pairs are shown. CTL, non-challenge control; CP, *C. perfringen*s-challenged group; ARGCP, *C. perfringens*-challenged group fed diet supplemented with 0.3% l-arginine.

*T*-test analysis was performed to evaluate the differentially abundant genera (relative abundance >0.1%) in Figure 3. A total of 24 taxa were significantly more abundant in the CP group than in the CTL group; among these taxa, 14 genera belonged to *Proteobacteria* (e.g., *Nitrosomonas* spp., *Coxiella* spp., *Ruegeria* spp., and *Thauera* spp., *P* < 0.05), 4 to *Planctomycetes* (e.g., *Planctomyces* spp. and *Blastopirellula* spp., *P* < 0.05), 3 to *Bacteroidetes* (*Phaeodactylibacter* spp., *Lutimonas* spp., and *Terrimonas* spp., *P* < 0.05), 1 to *Nitrospirae*, 1 to *Actinobacteria*, and 1 to *Acidobacteria* (Figure 3A and Table S3). However, compared with the CP group, ARGCP group had significantly less of most of these genera (Figure 3B, *P* < 0.05, except for *Caulobacter* spp.). In addition, the relative abundance of *Pseudomonas* spp. in the ARGCP group was significantly lower than that in the CP group (Figure 3B, *P* < 0.05). As shown in Figure 3C, the relative abundances of only three genera were significantly different between the CTL and ARGCP groups (*P* ≤ 0.001). The relative abundance of *Candidatus jettenia* spp. in the CTL group was significantly higher than that in the ARGCP group (*P* = 0.001), whereas *Thauera* spp. and *Gaiella* spp. were more abundant in the ARGCP group than in the control group (*P* < 0.001).

### Differentially abundant gut bacterial species

**Figure 4:**
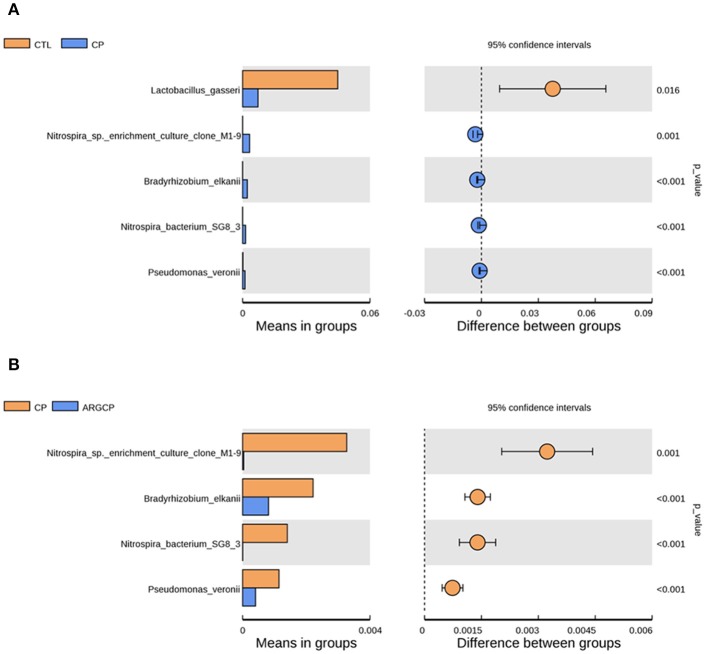
The species differentially abundant between three treatments in the ileum by *t*-test analysis. These figures illustrate the differences in the microbiota in terms of the relative abundances of species between groups CTL and CP **(A)**, and groups CP and ARGCP **(B)**. Only data whose differences with *P*-values lower than 0.05 and the relative abundances higher than 0.1% in either of the pairs are shown. CTL, non-challenge control; CP, *C. perfringen*s-challenged group; ARGCP, *C. perfringens*-challenged group fed diet supplemented with 0.3% l-arginine. There was no differentially abundant species between CTL group and ARGCP group.

At the species level, the relative abundance lower than 0.1% in all groups was filtered. The relative abundance of *Lactobacillus gasseri* dramatically decreased in CP group when compared to CTL group (Figure 4A, *P* < 0.05). Furthermore, the relative abundance of four bacterial species including *Nitrospira* sp. *enrichment culture clone* M1-9, *Bradyrhizobium elkanii, Nitrospira bacterium* SG8-3, and *Pseudomonas veronii* was significantly increased by the *C. perfringens* challenge (Figure 4A, *P* ≤ 0.001). However, the increases of the relative abundance of the above four bacterial species were significantly reduced by l-arginine supplementation (Figure 4B, *P* ≤ 0.001). There was no differentially abundant species between CTL group and ARGCP group (*P* > 0.05).

### Predicted functional changes in gut microbial communities

**Figure 5:**
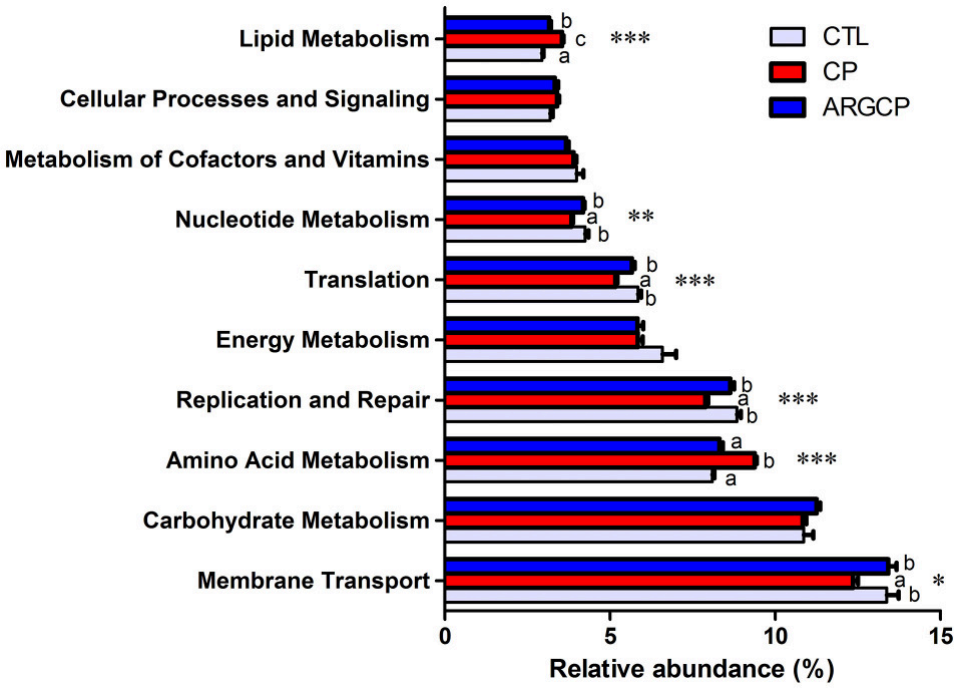
The 10 most abundant microbial pathways grouped into level-2 functional categories using PICRUSt. CTL, non-challenge control; CP, *C. perfringen*s-challenged group; ARGCP, *C. perfringens*-challenged group fed diet supplemented with 0.3% l-arginine. Different lowercase letters at each column indicate significant differences. Values are means with their standard errors. *P < 0.05, **P < 0.01, ***P < 0.001.

Alterations in the presumptive functions of the ileal microbiota of broilers were evaluated using PICRUSt analysis. Figure 5 shows the top 10 predicted microbial functions at level 2 of the KEGG pathways. Compared with CTL group, the CP group had significantly less abundance of KEGG pathways affiliated with membrane transport, replication and repair, translation and nucleotide metabolism (*P* < 0.05) and remarkably larger abundances of KEGG pathways belonging to amino acid metabolism and lipid metabolism (*P* < 0.05). l-arginine significantly reversed the changes in the abundances of these pathways (*P* < 0.05). A predicted microbial function comparison at level 3 of the KEGG pathways is shown in Table 5. Compared with the CTL group, the CP group had significantly lower abundance of 19 functional pathways, including two pathways in membrane transport (“Transporters” and “Phosphotransferase system”), five pathways in carbohydrate metabolism (e.g., “Amino sugar and nucleotide sugar metabolism” and “Glycolysis/Gluconeogenesis”), five pathways in replication and repair (e.g., “DNA repair and recombination proteins” and “Chromosome”), two pathways in energy metabolism (“Photosynthesis proteins” and “Photosynthesis”), three pathways in translation (e.g., “Ribosome” and “Ribosome Biogenesis”) and two pathways in nucleotide metabolism (“Purine metabolism” and “Pyrimidine metabolism”), while most of these changes were reversed by l-arginine supplementation (*P* < 0.05, except “Photosynthesis proteins” and “Photosynthesis”). Three pathways in carbohydrate metabolism (e.g., “Pyruvate metabolism” and “Butanoate metabolism”), two pathways in amino acid metabolism (“Arginine and proline metabolism” and “Glycine, serine and threonine metabolism”) and two pathways in energy metabolism (“Oxidative phosphorylation” “Carbon fixation pathways in prokaryotes”) were represented more in the CP group than in the CTL group (*P* < 0.05), whereas the ARGCP group had significantly less abundance of these pathways than the CP group (*P* < 0.05). Table 5 illustrated that in l-arginine-fed broilers, the relative abundances of KEGG pathways at level 3 were almost the same as those of the controls (except for 8 microbial functions whose abundances differed between the CTL and ARGCP groups).

**Table 5:** The selected main microbial pathways grouped into level-3 functional categories using PICRUSt.

**KEGG pathways**	**CTL**	**CP**	**ARGCP**	***P*-value**
**MEMBRANE TRANSPORT**				
Transporters	6.99 ± 1.20^b^	6.18 ± 0.08^a^	6.95 ± 0.09^b^	0.001
ABC transporters	3.32 ± 0.04	3.22 ± 0.06	3.28 ± 0.08	0.505
Secretion system	1.41 ± 0.02	1.55 ± 0.03	1.44 ± 0.06	0.051
Phosphotransferase system (PTS)	1.10 ± 0.13^b^	0.75 ± 0.04^a^	1.19 ± 0.04^b^	0.004
**CARBOHYDRATE METABOLISM**				
Amino sugar and nucleotide sugar metabolism	1.58 ± 0.05^b^	1.36 ± 0.03^a^	1.60 ± 0.04^b^	0.001
Glycolysis/Gluconeogenesis	1.36 ± 0.03^b^	1.22 ± 0.02^a^	1.34 ± 0.02^b^	0.002
Fructose and mannose metabolism	1.16 ± 0.06^ab^	1.04 ± 0.08^a^	1.33 ± 0.07^b^	0.033
Pyruvate metabolism	1.10 ± 0.01^a^	1.14 ± 0.01^b^	1.11 ± 0.01^a^	0.013
Starch and sucrose metabolism	1.10 ± 0.04^b^	0.86 ± 0.01^a^	1.05 ± 0.02^b^	< 0.001
Pentose phosphate pathway	0.91 ± 0.01^c^	0.78 ± 0.01^a^	0.88 ± 0.01^b^	< 0.001
Butanoate metabolism	0.61 ± 0.02^a^	0.90 ± 0.02^c^	0.75 ± 0.01^b^	< 0.001
Galactose metabolism	0.75 ± 0.05^b^	0.56 ± 0.02^a^	0.73 ± 0.02^b^	0.001
Propanoate metabolism	0.52 ± 0.02^a^	0.79 ± 0.01^c^	0.60 ± 0.01^b^	< 0.001
**AMINO ACID METABOLISM**				
Amino acid related enzymes	1.36 ± 0.01	1.32 ± 0.01	1.35 ± 0.02	0.151
Arginine and proline metabolism	1.01 ± 0.03^a^	1.09 ± 0.01^b^	0.98 ± 0.02^a^	0.006
Alanine, aspartate and glutamate metabolism	0.86 ± 0.01	0.89 ± 0.01	0.87 ± 0.02	0.333
Cysteine and methionine metabolism	0.83 ± 0.01	0.81 ± 0.01	0.84 ± 0.01	0.251
Glycine, serine and threonine metabolism	0.67 ± 0.01^a^	0.83 ± 0.01^c^	0.70 ± 0.004^b^	< 0.001
Lysine biosynthesis	0.67 ± 0.01	0.65 ± 0.01	0.67 ± 0.02	0.380
**REPLICATION and REPAIR**				
DNA repair and recombination proteins	2.89 ± 0.03^b^	2.59 ± 0.02^a^	2.82 ± 0.04^b^	< 0.001
Chromosome	1.59 ± 0.01^b^	1.44 ± 0.02^a^	1.56 ± 0.02^b^	< 0.001
DNA replication proteins	1.17 ± 0.02^b^	1.01 ± 0.01^a^	1.14 ± 0.02^b^	< 0.001
Homologous recombination	0.92 ± 0.01^c^	0.79 ± 0.01^a^	0.88 ± 0.01^b^	< 0.001
Mismatch repair	0.81 ± 0.01^b^	0.70 ± 0.01^a^	0.78 ± 0.01^b^	< 0.001
**ENERGY METABOLISM**				
Oxidative phosphorylation	1.05 ± 0.06^a^	1.18 ± 0.02^b^	0.98 ± 0.02^a^	0.008
Methane metabolism	1.06 ± 0.02	1.06 ± 0.01	1.04 ± 0.02	0.452
Carbon fixation pathways in prokaryotes	0.82 ± 0.01^a^	0.97 ± 0.01^c^	0.84 ± 0.01^b^	< 0.001
Photosynthesis proteins	1.12 ± 0.17^b^	0.57 ± 0.07^a^	0.76 ± 0.07^a^	0.011
Photosynthesis	0.94 ± 0.13^b^	0.50 ± 0.05^a^	0.66 ± 0.06^a^	0.008
**TRANSLATION**				
Ribosome	2.41 ± 0.03^b^	2.09 ± 0.03^a^	2.32 ± 0.04^b^	< 0.001
Ribosome Biogenesis	1.44 ± 0.04^b^	1.31 ± 0.01^a^	1.43 ± 0.01^b^	0.002
Aminoacyl-tRNA biosynthesis	1.25 ± 0.02^b^	1.11 ± 0.02^a^	1.19 ± 0.03^b^	0.001
**NUCLEOTIDE METABOLISM**				
Purine metabolism	2.40 ± 0.05^b^	2.24 ± 0.03^a^	2.40 ± 0.02^b^	0.007
Pyrimidine metabolism	1.84 ± 0.05^b^	1.60 ± 0.02^a^	1.79 ± 0.02^b^	0.001

a, b, c *Values within a column followed by different superscript letters differ significantly (P < 0.05). CTL, non-challenge control; CP, C. perfringens-challenged group; ARGCP, C. perfringens-challenged group fed diet supplemented with 0.3% l-arginine. Data are shown as the mean ± standard error*.

## Discussion

A surge of information regarding the gut microbiota and its impacts on health has been reported over the past decade. The gut microbial community structure is strongly affected by many factors such as diet, age, immunological response, host genotype, antibiotic addition, and pathogen infection (Luo et al., 2017). Previous researches have demonstrated that diet has a great influence (estimated at 57%, compared with 12% for genetic factors) on the gut microbial community structure (Tomasello et al., 2014). The community structure of microbiota can be quickly altered by the changes of dietary components (David et al., 2014). l-arginine possesses potential benefits for both humans and animals against numerous diseases, such as alleviating necrotizing enterocolitis of premature neonates and coccidiosis and infectious bursal disease of broiler chickens (Amin et al., 2002; Tan et al., 2014a, 2015). In our previous studies, Dietary l-arginine supplementation protected the gut mucosa by improving innate immune responses, intestinal absorption and barrier function and by suppressing the colonization of *C. perfringens* in necrotic enteritis-challenged broiler chickens (Zhang et al., 2017). However, little is known regarding how l-arginine influences the intestinal microbiota in broiler chickens.

In this experiment, all of the *C. perfringens-*challenged birds appeared obvious pathological changes in the small intestines such as hyperemia, small red petechiae, thinner walls and gas production in the intestinal tract, which has been documented by other researchers (Van Immerseel et al., 2004, 2009; Dahiya et al., 2005; Liu et al., 2010, 2012). Hematoxylin-eosin staining showed that *C. perfringens* challenge led to pathological changes and the disappearance of normal villus architecture (Figure 1). Also, previous studies of our lab reported that *C. perfringens* challenge impaired growth performance and led to mucosal atrophy and increased plasma endotoxin levels (Liu et al., 2012; Guo et al., 2014). All these data proved that the experimental NE model was successfully established. Moreover, l-arginine supplementation played a beneficial role in *C. perfringens*-challenged birds, as characterized by decreased gut lesion scores, enhanced villus height and preserved gut morphology.

The community structure of intestinal flora is relatively stable and similar in healthy hosts while intestinal microbiota disturbance can cause many diseases (Karlsson et al., 2013). The result of alpha diversity in our study showed that *C. perfringens* challenge significantly increased the richness and diversity of ileal microbiota (Table 2), which was also proven by Xu et al. (2018). Nevertheless, Li et al. (2017) reported that *C. perfringens* challenge dramatically reduced the observed species, Chao1 and ACE indices of the ileal microbiota. The discrepancy may be attributed to the different sections of ileum in which the digesta was collected. Here, the *C. perfringens* challenge-induced increases of microbial richness and diversity were reversed by l-arginine supplementation (Table 2), which reflected that l-arginine could prevent the intestinal flora disorder.

PCA and ANOSIM tests showed that bacterial communities varied across the different treatments (Figure 2 and Table 3). PCA indicated that the composition of the microbiota in the ARGCP group was more similar to that in the CTL group than that in the CP group (Figure 2), which might be due to the regulation of l-arginine. *Firmicutes, Cyanobacteria, Proteobacteria* and *Bacteroidetes* were the four pre-dominant bacterial taxa at the phylum level in the ileal microbiota (Table 4), which is similar to previous studies of broilers (Li et al., 2017; Wang et al., 2017). It has been reported that *Firmicutes* improved the utilization of energy in the diet and the ratio of *Firmicutes* to *Bacteroides* was often positively associated with weight gain (Ley et al., 2006; Bervoets et al., 2013). In the current study, the relative abundance of *Firmicutes* and the ratio of *Firmicutes* to *Bacteroides* were remarkably higher in the ARGCP group than in the CP group (Table 4 and Figure S4), which may be correlated with the effect of l-arginine promoting broiler growth performance (Tan et al., 2014a,b). In this research, *Proteobacteria* was more abundant in the CP group than in the CTL group (Table 4). Gut bacteria belonging to *Proteobacteria* include a wide variety of pathogens such as *Rickettsia* spp. (Ferla et al., 2013), *Brucella* spp. (de Figueiredo et al., 2015) and *Neisseria* spp. (Criss and Seifert, 2012). However, significantly less *Proteobacteria* was observed in the ARGCP group than in the CP group (Table 4), which was consistent with reports that l-arginine treatment reduced the number of harmful bacteria belonging to the phylum *Proteobacteria*, such as *Escherichia coli* (Liu et al., 2017) and *Helicobacter pylori* (Chaturvedi et al., 2007). Moreover, *C. perfringens* challenge resulted in significantly higher relative abundances of *Actinobacteria, Planctomycetes, Verrucomicrobia*, and *Chloroflexi* in the CP group than in unchallenged birds (Table 4). In some pathological cases such as cardiovascular diseases, non-alcoholic fatty liver disease and ulcerative colitis, the relative abundance of *Actinobacteria* was higher in patients than in healthy individuals (Lepage et al., 2011; Dinakaran et al., 2014; Del Chierico et al., 2017). In addition, *Verrucomicrobia* and *Chloroflexi*, despite usually being present in low abundance (a few percent or less), are consistent components in most individuals and animals, and their levels become elevated in some diseases (Abusleme et al., 2013; Campbell et al., 2014). *Planctomycetes* is phylogenetically closely related to *Verrucomicrobia* and *Chlamydiae* and widely distributed in vast anoxic zones of the ocean (Fuerst and Sagulenko, 2011). *Planctomycetes* spp. are major participants in the global nitrogen cycle since 50% of the nitrogen molecules in the atmosphere are generated by this genus (Fuerst and Sagulenko, 2011). However, the role that *Planctomycetes* plays in animals is unclear. The alterations of the gut microbiota community by l-arginine treatment in our research were associated with recovery from intestinal injury, suggesting that l-arginine-mediated reduction of the *Proteobacteria, Actinobacteria, Verrucomicrobia, Chloroflexi*, and *Planctomycetes* abundances played a benign role in the development of necrotic enteritis.

In the *t*-test analysis at the genus level, more than half of the genera differentially abundant between the three treatments belonged to the phylum *Proteobacteria* (Figure 3 and Table S3). In this phylum, the genera of *Nitrosomonas, Coxiella, Ruegeria*, and *Thauera* were more dominant in the CP group than in other groups (Figures 3A,B), and *Nitrosomonas* spp. were the most abundant species. Nitrification is conducted by two different types of bacteria. The first type are the ammonia-oxidizing bacteria, which oxidize ammonia to nitrite. The other type, the nitrite-oxidizing bacteria, convert nitrite to nitrate (Chauret et al., 2008). *Nitrosomonas*, a major genus of the ammonia-oxidizing bacteria, utilizes ammonia as its sole energy source (Lek Noophan et al., 2009). l-arginine addition inhibits the nitrite formation and protein synthesis of *Nitrosomonas europaea in vitro* (Clark and Schmidt, 1967). In our study, l-arginine treatment decreased the population of ammonia oxidizers, which might be due to different C and N availability, intrinsic amino acid characteristics and interactions with the host and heterotrophic microorganisms (Tzanakakis and Paranychianakis, 2017). Consistent with the changes in *Nitrosomonas* spp., the abundance of *Unidentified_Nitrospiraceae*, a genus of nitrite-oxidizing bacteria, was also markedly lower in the ARGCP group than in the CP group (Figure 3B). This result may reflect the synergistic efforts of ammonia-oxidizing bacteria and nitrite-oxidizing bacteria in nitrification. *Coxiella* spp. include different species of bacteria that use ticks as effective vectors to spread a range of disease (Khoo et al., 2016; Machado-Ferreira et al., 2016). A typical example is *Coxiella burnetii*, which is the causative agent for Q fever, an infectious disease with a global distribution commonly affecting animals and human (Khoo et al., 2016). Members of *Ruegeria* were linked to yellow band disease in Fungiidae corals (Apprill et al., 2013). The bacterium *Ruegeria pomeroyi* is more abundant in corals with white patch syndrome or skeletal growth anomalies than in healthy corals (Sere et al., 2013; Li et al., 2015). The genus *Thauera*, of the class *Betaproteobacteria*, has been detected in sediments, sludge, soils, and wastewater treatment plants (Liu et al., 2013), and it has been recently reported in research on human (Smith et al., 2016; Xu et al., 2017). Xu et al. (2017) observed that *Thauera* was significantly more abundant in individuals with substance use disorders than in healthy individuals. Similar to the result of Xu et al. (2017), *Thauera* was represented more in the CP group than in the CTL group in our study, indicating that this genus may comprise opportunistic pathogens. However, determining the specific functions of these bacteria requires further testing and verification. Furthermore, the abundances of only 3 genera differed between the CTL group and the ARGCP group, and the abundances of most genera were not significantly different between these pairs (Figure 3C), which indicated that the diet supplemented with l-arginine normalized the ileal microbiota of broiler chickens challenged with *C. perfringens*, returning its microbial structure to resemble that of healthy controls, and that the beneficial effects of l-arginine on intestinal injury may be largely mediated by the composition of the gut microbiota.

*Lactobacillus gasseri* is one kind of the well-established probiotic microorganisms. In humans, *Lactobacillus gasseri* was shown to competitively inhibit the growth of harmful bacteria, produce bacteriocin and modulate the innate and adaptive systems, eliciting many health benefits (Selle and Klaenhammer, 2013). On the species level, *Lactobacillus gasseri* was less abundant in CP group than in CTL group (Figure 4A), which reflected that *C. perfringens* challenge inhibited the growth of some probiotic bacteria. In our study, *Nitrospira* sp. *enrichment culture clone* M1-9 and *Nitrospira bacterium* SG8-3, belonged to the genus of *unidentified_Nitrospiraceae*, were more abundant in CP group than other groups (Figures 4A,B). This was in accordance with our observation on the changes of the relative abundance of *unidentified_Nitrospiraceae* spp. *Pseudomonas veronii* has been found in natural mineral waters, soils and animal intestines. This bacterium was reported to be the aetiological agent of human intestinal inflammatory pseudotumour (Cheuk et al., 2000). Here, l-arginine supplementation reversed the increased relative abundance of *Pseudomonas veronii* caused by *C. perfringens* challenge. This suggested that l-arginine supplementation could inhibit this potential opportunistic pathogen.

According to the predictive functional profiles of microbial communities determined by PICRUSt analysis, the most abundant functions were membrane transport, carbohydrate metabolism and amino acid metabolism (Figure 5), and clear differences were observed in the level 3 KEGG pathways between the three treatments (Table 5). Membrane transport pathways, such as those involving transporters and ABC transporters, are essential to cell viability and growth and therefore crucial for the survival of bacteria in the gut ecosystem (Lyons et al., 2017). Odamaki et al. (2016) demonstrated that such predicted transporter functions were connected with nutrient-associated changes in gut microbiota composition. In our study, l-arginine supplementation significantly increased the proportion of transporters, suggesting that l-arginine might play an important role in affecting the gut microbiota composition of birds challenged with *C. perfringens*. A phosphotransferase system (PTS) is used by bacteria for sugar uptake and uses phosphoenolpyruvate, a key intermediate in glycolysis, as the source of energy (Erni, 2013). PTS pathways are more abundant in the ARGCP group than in the CP group, which may be closely associated with the promotion of various sugar metabolism pathways (for instance, glycolysis/gluconeogenesis, fructose, mannose, sucrose, and galactose metabolisms) (Table 5). The results suggested that l-arginine could play an active role in the progress that intestinal microbiota sensed and utilized sugars as resources for the production of energy and synthesis of cellular components.

Our data showed the amino acid and lipid metabolisms were overrepresented in the ileal microbiota of challenged chickens (Figure 5). Similarly, according to the research of Davenport et al. (2014), bacterial functional pathways were notably altered in the inflamed tissue of ulcerative colitis patients, with decreased carbohydrate metabolism and promoted lipid and amino acid metabolisms. The author inferred that the supply of carbohydrates was reduced and that bacteria that metabolize amino acids and lipids dominate in the inflamed gut. However, in our study, carbohydrate metabolism was not affected by the three treatments. This result may be related to differences in the hosts and disease models used in the studies. The small intestine is rich in monosaccharides, disaccharides and amino acids, which supports the growth of certain bacteria, particularly *Proteobacteria* and *Lactobacillales* (Kamada et al., 2013). In our data, the reduction in amino acid metabolism may be closely related to the low abundance of *Proteobacteria* in the ARGCP group.

In this study, the *C. perfringen*s challenge enriched pathways related to arginine and proline metabolism and glycine, serine and threonine metabolism (Table 5). As summarized by previous reviews, arginine can be used as an energy source by various pathogens such as *Pseudomonas aeruginosa, Helicobacter pylori* and *Salmonella typhimurium* (Gogoi et al., 2016; Xiong et al., 2016). The bacteria degrade arginine and produce urea and ammonia, which can diminish the availability of arginine substrate for host-cell inducible nitric oxide synthase (iNOS), thereby reducing nitric oxide production and facilitating evasion from its antibacterial effects (Gobert et al., 2001). In this experiment, the acceleration of arginine metabolism by the gut microbiota in challenged birds may cause arginine deprivation in the host, leading to compromised T-cell function and eventually resulting in increased susceptibility to infection (Popovic et al., 2007). l-arginine supplementation might normalize arginine metabolism, which may result in that l-arginine significantly enhancing T-cell proliferation and function (Ochoa et al., 2001; Taheri et al., 2001; Tan et al., 2015) and thus inhibiting pathogens catabolizing l-arginine (Stadelmann et al., 2013). In the current experiment, l-arginine treatment depleted the pathways related to glycine, serine and threonine metabolism (Table 5). This finding was consistent with the results of Dai et al. (2012) and demonstrated that the net utilization of glycine, serine and threonine by mixed ileal bacteria was reduced with arginine addition *in vitro*. Recent research showed that the pathogenic bacteria *Staphylococcus aureus* utilizes serine and threonine for producing hemolysin, which contributes to the virulence of the bacteria (Burnside et al., 2010).

## Conclusion

In summary, we found that Dietary l-arginine supplementation promoted the intestinal health and modulated the ileal microbiota of broiler chickens challenged with *C. perfringens* to result in a consortium similar to that of healthy controls, characterized by enriched helpful bacteria and suppressed harmful species.

## Author contributions

YG and BZ designed the research. BZ conducted the animal feeding. BZ, ZPL, and GL performed sample collection and detection. BZ, ZL, and WW analyzed the data. BZ and YG wrote the manuscript. All authors approved the final manuscript.

### Conflict of interest statement

The authors declare that the research was conducted in the absence of any commercial or financial relationships that could be construed as a potential conflict of interest.
