# Supplementary material for: Dietary l-arginine Supplementation Alleviates the Intestinal Injury and Modulates the Gut Microbiota in Broiler Chickens Challenged by Clostridium perfringens
Source: Front Microbiol. 2018 Jul 31;9:1716. doi: 10.3389/fmicb.2018.01716 (PMC6080643; doi:10.3389/fmicb.2018.01716)
Supplement: Supplementary file 1 [file Data_Sheet_1.docx]

Supplementary Material

Dietary l-Arginine Supplementation Alleviates the Intestinal Injury and Modulates the Gut microbiota in Broiler Chickens Challenged by *Clostridium perfringens*

**Beibei Zhang, Zengpeng Lv, Zhui Li, Weiwei Wang, Guang Li，Liping Gan and Yuming Guo***

State Key Laboratory of Animal Nutrition, College of Animal Science and Technology, China Agricultural University, Beijing, China

*** *Correspondence:** Corresponding Author**:** [guoyum@cau.edu.cn](mailto:guoyum@cau.edu.cn)

1. **Supplementary Figures and Tables**
   1. **Supplementary Figures**

**Figure S1. Venn diagram of the OTUs.** CTL, non-challenge control; CP, *C. perfringens*-challenged group; ARGCP, *C. perfringen*s-challenged group fed diet containing 0.3% l-arginine.

**
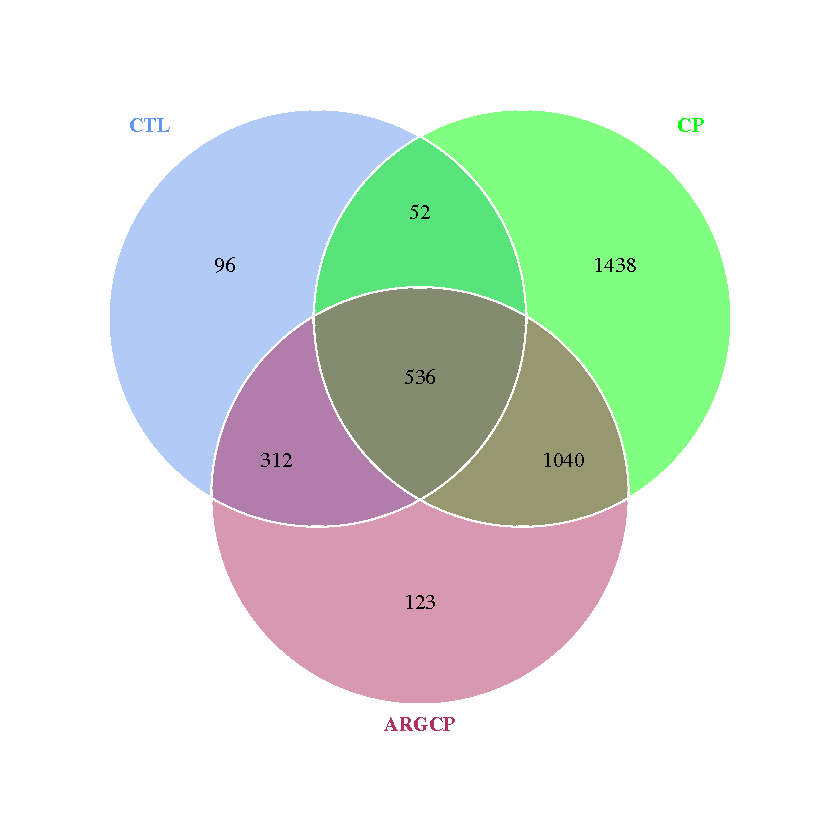
**

**Figure S2. The rarefaction curve analysis of the microbial species.** CTL, non-challenge control; CP, *C. perfringens*-challenged group; ARGCP, *C. perfringen*s-challenged group fed diet containing 0.3% l-arginine.

**
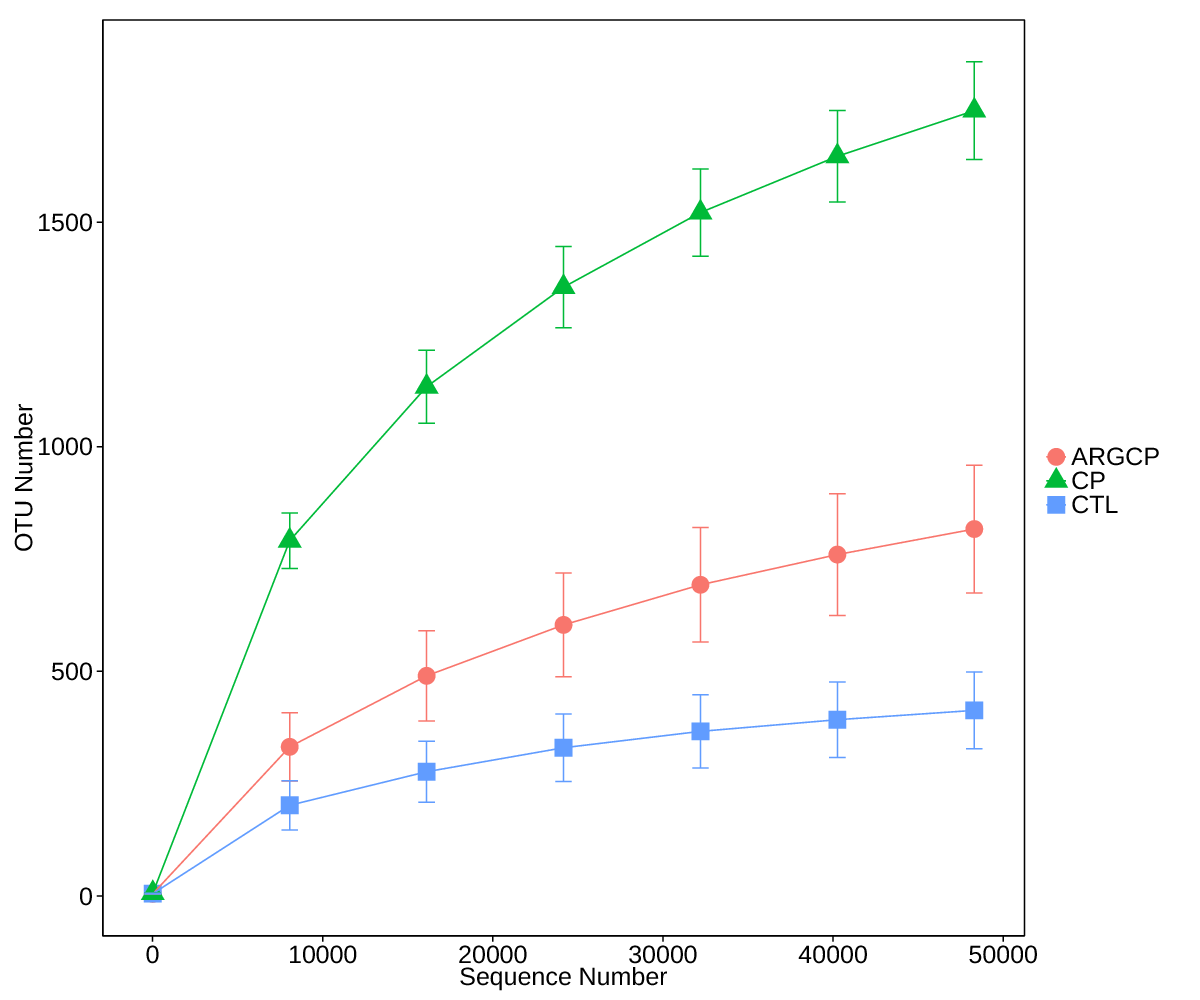
**

**Figure S3. Relative abundances of bacteria at the phylum level in different treatments.** CTL, non-challenge control; CP, *C. perfringens*-challenged group; ARGCP, *C. perfringen*s-challenged group fed diet containing 0.3% l-arginine.


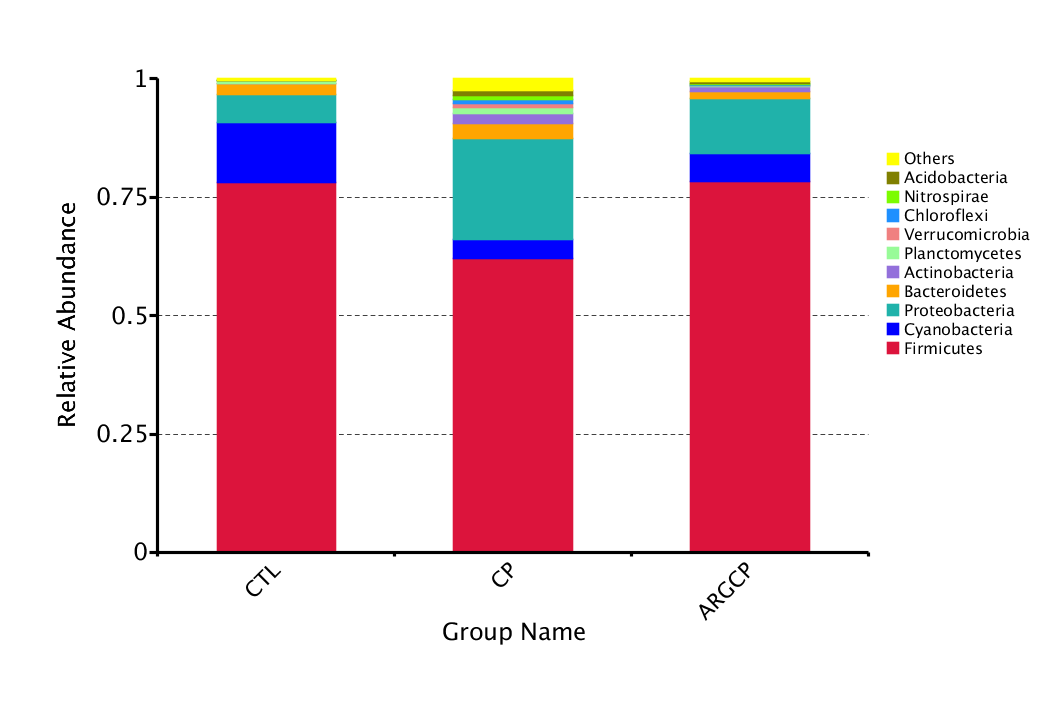


**Figure S4. Ratios of *Firmicutes* abundance to *Bacteroidetes* abundance in the ileal microbiota of broilers.** CTL, non-challenge control; CP, *C. perfringens*-challenged group; ARGCP, *C. perfringen*s-challenged group fed diet containing 0.3% l-arginine. Statistical significance was set at *P* < 0.05. Values are means with their standard errors.


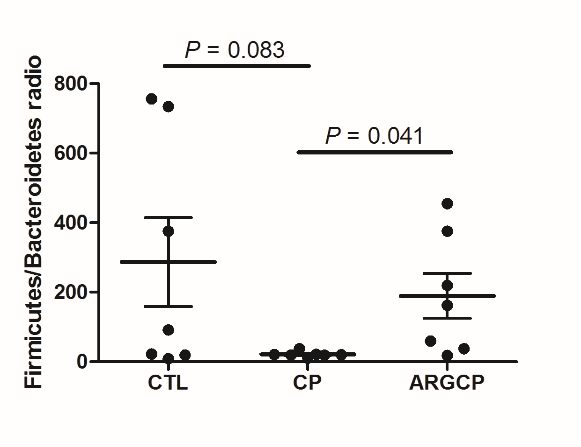


- 1. **Supplementary Tables**

**Table S1. Composition of the diet and nutrient levels.** ^a^Supplied per kilogram of complete feed: Mn, 100 mg; Fe, 80 mg; Zn, 75 mg; Cu, 8 mg; I, 0.35 mg; and Se, 0.15 mg. ^b^Supplied per kilogram of complete feed: vitamin A, 12,500 IU; vitamin D_3_, 2,500 IU; vitamin E, 30 IU; vitamin K_3_, 2.65 mg; vitamin B_1_, 2 mg; vitamin B_2_, 6 mg; vitamin B_5_, 12 mg; vitamin B_12_, 0.025 mg; niacin, 50 mg; folic acid, 1.25 mg and biotin, 0.0325 mg. ^c^Calculated composition, unless otherwise indicated.

| Item (%, unless otherwise indicated) | Control diet | l-arginine-supplemented diet |
| --- | --- | --- |
| Ingredient |  |  |
| Corn | 58.28 | 58.28 |
| Soybean meal (44% CP) | 29.11 | 29.11 |
| Corn gluten meal | 5.00 | 5.00 |
| Soybean oil | 2.50 | 2.50 |
| Dicalcium phosphate | 1.92 | 1.92 |
| Limestone | 0.89 | 0.89 |
| Sodium chloride | 0.30 | 0.30 |
| Choline chloride (50%) | 0.25 | 0.25 |
| Mineral premix^a^ | 0.20 | 0.20 |
| D,L-Methionine (98%) | 0.26 | 0.26 |
| L-Lys-HCl (98%) | 0.24 | 0.24 |
| Vitamin premix^b^ | 0.03 | 0.03 |
| Ethoxyquin (33%) | 0.02 | 0.02 |
| L-Arg | 0.00 | 0.40 |
| L-Ala | 1.00 | 0.60 |
| Nutrient level^c^ |  |  |
| ME (Mcal/kg) | 2.98 | 2.98 |
| CP | 21.94 | 21.94 |
| Ca | 1.00 | 1.00 |
| Non-phytate phosphorus | 0.45 | 0.45 |
| Lys (analyzed value) | 1.36 | 1.39 |
| Met | 0.59 | 0.59 |
| Thr (analyzed value) | 0.86 | 0.88 |
| Arg (analyzed value) | 1.42 | 1.72 |

**Table S2. Sequencing depth.** CTL, non-challenge control; CP, *C. perfringen*s-challenged group; ARGCP, *C. perfringens*-challenged group fed diet supplemented with 0.3% l-arginine.

| Sample name | Effective tags | Average length (bp) | Good's coverage (%) |
| --- | --- | --- | --- |
| CTL1 | 72,857 | 253 | 99.8 |
| CTL2 | 80,703 | 253 | 99.7 |
| CTL3 | 60,652 | 253 | 99.8 |
| CTL4 | 49,693 | 255 | 99.9 |
| CTL5 | 61,903 | 253 | 99.8 |
| CTL6 | 75,731 | 253 | 99.7 |
| CTL7 | 48,955 | 253 | 99.7 |
| CP1 | 88,147 | 253 | 98.7 |
| CP2 | 77,138 | 253 | 98.9 |
| CP3 | 79,756 | 254 | 98.8 |
| CP4 | 83,539 | 254 | 98.7 |
| CP5 | 72,464 | 253 | 99.0 |
| CP6 | 74,240 | 253 | 99.1 |
| CP7 | 76,599 | 254 | 99.0 |
| ARGCP1 | 77,655 | 253 | 99.4 |
| ARGCP2 | 85,574 | 253 | 99.2 |
| ARGCP3 | 63,604 | 253 | 99.5 |
| ARGCP4 | 82,264 | 253 | 99.5 |
| ARGCP5 | 76,549 | 253 | 99.4 |
| ARGCP6 | 69,154 | 254 | 99.4 |
| ARGCP7 | 81,430 | 253 | 99.2 |

**Table S3.** **Bacterial taxonomy of the genera differentially abundant between three treatments.**

| Phylum | Class | Family | Genus |
| --- | --- | --- | --- |
| *Proteobacteria* | *Alphaproteobacteria* | *Bradyrhizobiaceae* | *Bradyrhizobium* |
|  | *Alphaproteobacteria* | *Caulobacteraceae* | *Caulobacter* |
|  | *Alphaproteobacteria* | *Phyllobacteriaceae* | *Mesorhizobium* |
|  | *Alphaproteobacteria* | *Rhodobacteraceae* | *Paracoccus* |
|  | *Alphaproteobacteria* | *Rhodobacteraceae* | *Ruegeria* |
|  | *Alphaproteobacteria* | *Rhodospirillaceae* | *Magnetovibrio* |
|  | *Alphaproteobacteria* | *Rhodospirillaceae* | *Pelagibius* |
|  | *Betaproteobacteria* | *Nitrosomonadaceae* | *Nitrosomonas* |
|  | *Betaproteobacteria* | *Rhodocyclaceae* | *Thauera* |
|  | *Deltaproteobacteria* | *Bdellovibrionaceae* | *Bdellovibrio* |
|  | *Deltaproteobacteria* | *Desulfurellaceae* | *H16* |
|  | *Gammaproteobacteria* | *Coxiellaceae* | *Coxiella* |
|  | *Gammaproteobacteria* | *Halieaceae* | *Haliea* |
|  | *Gammaproteobacteria* | *Halieaceae* | *Halioglobus* |
|  | *Gammaproteobacteria* | *Pseudomonadaceae* | *Pseudomonas* |
| *Planctomycetes* | *Planctomycetacia* | *Brocadiaceae* | *Candidatus_jettenia* |
|  |  | *Phycisphaeraceae* | *SM1A02* |
|  |  | *Planctomycetaceae* | *Blastopirellula* |
|  |  | *Planctomycetaceae* | *Pir4_lineage* |
|  |  | *Planctomycetaceae* | *Planctomyces* |
| *Bacteroidetes* | | *Chitinophagaceae* | *Terrimonas* |
|  |  | *Flavobacteriaceae* | *Lutimonas* |
|  |  | *Saprospiraceae* | *Phaeodactylibacter* |
| *Nitrospirae* | | *Nitrospiraceae* | *unidentified_Nitrospiraceae* |
| *Actinobacteria* | | *Gaiellaceae* | *Gaiella* |
| *Acidobacteria* | | *Blastocatellaceae_(Subgroup_4)* | *RB41* |

**
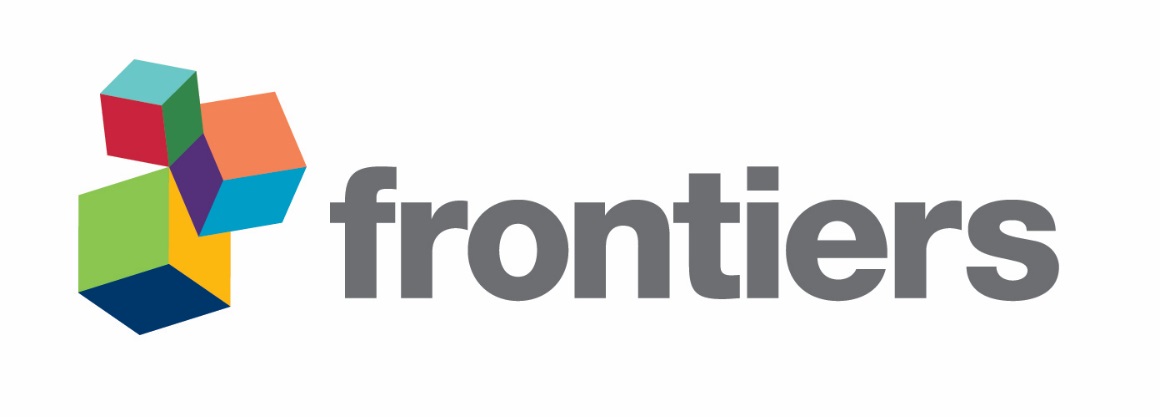
**
